# Supplementary material for: Indirect Treatment Comparison of Larotrectinib versus Entrectinib in Treating Patients with TRK Gene Fusion Cancers
Source: Cancers (Basel). 2022 Mar 31;14(7):1793. doi: 10.3390/cancers14071793 (PMC8997457; doi:10.3390/cancers14071793)
Supplement: Supplementary file 1 [file cancers-14-01793-s001.zip › cancers-1618439-supplementary.pdf]

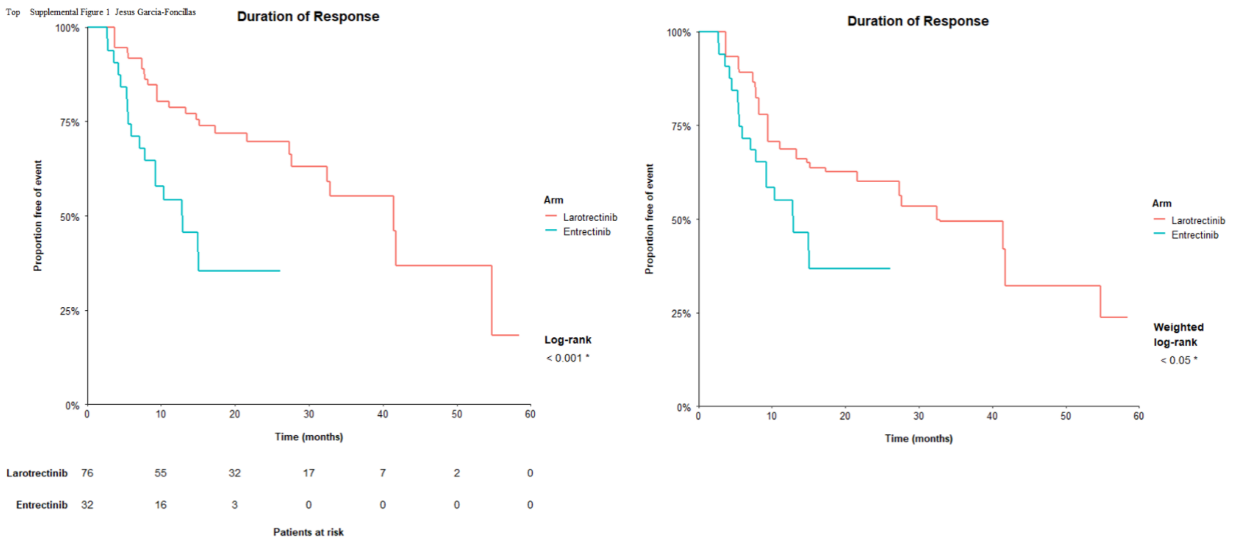

Supplemental Figure 1. Duration of response with larotrectinib vs. entrectinib. (A) Kaplan-Meier curve for duration of response before matching, (B) Kaplan-Meier curve for duration of response after matching on primary matching factors [a]. Notes: [a.] Primary matching variables include: male, age above median in entrectinib population in Rolfo,[9] 2020 (>57 years), White, Black, Asian, ECOG PS score 0, ECOG PS score 1, tumor (thyroid), tumor (sarcoma), tumor (salivary), tumor (lung), metastatic disease (vs. locally advanced, unresectable disease), central nervous system metastases (yes), NTRK1, NTRK2, prior lines of systemic therapy for metastatic disease (0), prior lines of systemic therapy for metastatic disease (1), prior lines of systemic therapy for metastatic disease (2). * denotes statistical significance (alpha <0.05)
